# The WHO checklist: a global tool to prevent errors in surgery

**DOI:** 10.1186/1754-9493-3-9

**Published:** 2009-05-28

**Authors:** Sukhmeet S Panesar, Kevin Cleary, Aziz Sheikh, Liam Donaldson

**Affiliations:** 1 National Patient Safety Agency (NPSA), Patient Safety Division, 4-8 Maple Street, London, W1T 5HD, UK; 2 Division of Community Health Sciences: GP Section, University of Edinburgh, 20 West Richmond Street, Edinburgh, EH8 9DX, UK; 3 Department of Health Richmond House 79 Whitehall, London, SW1A 2NS, UK

## Abstract

In this article, we welcome the adoption of the WHO surgical checklist to prevent errors in surgical practice. We highlight the scale of the problem and discuss the adoption of this tool in the UK.

## Background

The increased complexity of healthcare has led to a corresponding increase in the number of medical errors. A significant proportion (up to 10%) of hospitalized patients experience a patient safety incident and nearly half of these are preventable. [1] Numerically, this translates to just under 100,000 preventable patient deaths per year. [2] Approximately 1 in 8 British individuals have a surgical procedure performed each year; [3] these typically bringing them considerable benefits, but also subjecting them to significant risk of potentially avoidable harm.

Significant advances have been made internationally through the World Health Organization's World Alliance for Patient Safety and through legislation to focus increased attention on patient safety considerations. One of the areas of particularly high priority is the creation of patient safety reporting systems which aim to help identify patterns of errors and through so doing facilitate learning and the formulation of harm reduction strategies. [4]

**Figure 1:**
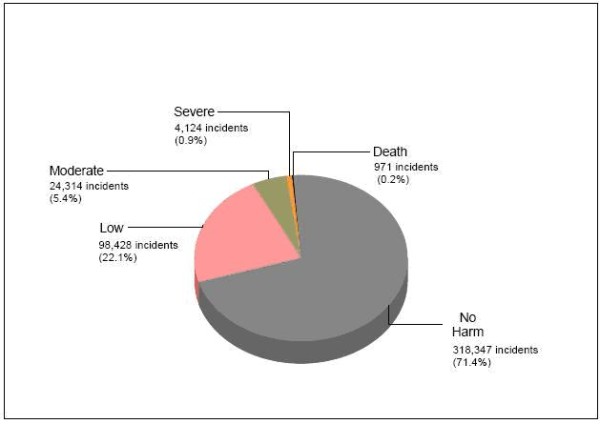
**Degree of harm for surgical incidents occurring in the Reporting and Learning System (RLS) at the NPSA between January 2005 and September 2008**.

The UK has been spearheading the patient safety agenda and is a pioneer in developing the first national repository of patient safety events i.e. the Research and Learning Service (RLS) database, which is maintained by the National Patient Safety Agency (NPSA). This is now the largest database of patient safety incidents in the world. These incidents are arranged categorically. To date, the NPSA has received in excess of 3 million reports [5] of which 450,000 are surgically-related (see Figure 1).

The recently launched WHO Surgical Checklist is an important development, which may help to prevent a number of these surgical errors. Encouragingly, it has now been adapted for use in England and Wales. [6]

One of the key error-prone areas that the surgical checklist [7] can mitigate against is that of 'Wrong-Site Surgery.' Wrong site or wrong patient incidents are rare, but the consequences can result in considerable harm to the patient. A recent study revealed 5,940 cases of wrong-site surgery (2,217 wrong side surgical procedures and 3,723 wrong-treatment/wrong procedure errors) in 13 years. [8] Our review of the RLS database (September 2007 – August 2008) revealed 26 (3.6%) cases of wrong patient, 62 (8.5%) of wrong side block, 150 (20.7%) of wrong side marked on consent form, 78 (10.7%) of wrong side marked on patient, 353 (48.6%) of wrong side marked on theatre list, 11 (1.5%) of wrong site prosthesis and 46 (6.3%) of wrong side surgery. These results are likely to be a gross under-representation of the true number of these events as reporting to the RLS is still far from complete. [9]

The important study by Haynes et al. [6] has demonstrated that use of a simple checklist can substantially and significantly reduce risk of morbidity and mortality associated with surgery, and given the importance of this finding in a field that tends to be characterised by relatively little in the way of robust evidence, we have taken the policy decision to nationally implement routine use of this approach. [10] Over the next year we expect all National Health Service trusts to have adopted this very simple and effective intervention.

## Competing interests

The authors declare that they have no competing interests.

## Authors' contributions

SSP contributed to conception, design, analysis, interpretation of data, and drafted the manuscript. KC, AS and LD were involved in analysis and interpretation of data and revised the manuscript critically for important intellectual content. All authors read and approved the final manuscript.

## Authors' information

SSP is a clinical advisor to the Medical Director, National Patient Safety Agency (NPSA), KC is the Medical Director, NPSA, AS is Professor of Primary Care, Research and Development, University of Edinburgh and LD is the Chief Medical Officer for England.
